# Obesity as an additional factor for autonomic imbalance and poor sleep behavior in chronic obstructive pulmonary disease: a case-control study

**DOI:** 10.6061/clinics/2021/e1826

**Published:** 2021-01-11

**Authors:** Cristiano Mostarda, Catarina de Andrade Barboza, Ana Lídia Cutrim, Antonio Carlos Silva-Filho, Carlos José Dias, Janaina De Oliveira Brito-Monzani, Bruno Rodrigues

**Affiliations:** I Laboratorio de Adaptacoes Cardiovasculares ao Exercicio (LACORE), Universidade Federal do Maranhao, Sao Luis, MA, BR; II Programa de Pos-Graduacao em Educacao Fisica, Universidade Federal do Maranhao, Sao Luis, MA, BR; III Centro de Reabilitacao Cardiopulmonar, Hospital Universitario “Presidente Dutra”, Universidade Federal do Maranhao, Sao Luis, MA, BR; IV Faculdade de Educacao Fisica, Universidade Estadual de Campinas (UNICAMP), Campinas, SP, BR

**Keywords:** Overweight, Chronic Obstructive Pulmonary Disease, Autonomic Modulation, Sleep Behavior

## Abstract

**OBJECTIVES:**

We compared the autonomic modulation and sleep behavior of eutrophic and overweight patients with chronic obstructive pulmonary disease (COPD).

**METHODS:**

COPD participants were divided into the overweight and eutrophic groups. Pulmonary function, blood pressure, body composition, autonomic modulation, and the Pittsburgh Sleep Quality Index score were assessed. Participants performed the six-minute walk test for functional assessment.

**RESULTS:**

Spirometric variables obtained in eutrophic and overweight individuals with COPD showed no statistically different results. We observed that the SDNN index indicated lower overall variability (*p*=0.003), and root mean square of successive differences between normal heart beats (RMSSD) (*p*=0.04) indicated lower parasympathetic modulation in the overweight group than observed in the eutrophic group. The indexes of the frequency domain presented lower values of total variability (*p*<0.01), low frequency bands (*p*<0.01), and high frequency (*p*=0.02), suggesting a higher sympathetic modulation and reduced parasympathetic modulation of the overweight group compared to eutrophic group. The overweight group also showed reduced sleep quality than the eutrophic group.

**CONCLUSION:**

Overweight COPD patients showed lower autonomic modulation and impaired sleep quality, latency, and efficiency as compared eutrophic subjects. These results reinforce the importance of weight control and the acquisition of healthy habits in this population.

## INTRODUCTION

Chronic obstructive pulmonary disease (COPD) is a chronic disease strongly associated with severe morbidity and is currently the third leading cause of death globally (1). Patients with COPD display airway inflammation and destruction of the lung parenchyma, mainly due to redox imbalance and proinflammatory cytokines (2). These changes induce small airway dysfunction limiting the flow and typically present symptoms such as cough and progressive chronic dyspnea, which are limiting conditions for patients (3).

Changes in respiratory function may directly influence the autonomic nervous system (ANS) adjustments, where the cardiovascular pace and control of the circadian cycle occur (4). Therefore, the impairment of sensitivity in autonomic reflexes, such as increased sympathetic tone and/or loss of parasympathetic tone, has been considered a significant risk factor for cardiac morbidity and mortality (5).

Additionally, changes in cardiac autonomic complexity are described as pathophysiological features of obesity (6), suggesting that cardiac autonomic dysfunction in obese patients is related to sympathetic hyperactivation and detriment of the vagus nerve (7). In fact, there is a direct association between higher blood pressure (BP) levels and worsening cardiac autonomic modulation in obese subjects (8). Therefore, risk factors such as overweight have alarmingly increased worldwide and are associated with severe morbidity and mortality (1). In the context of COPD, overweight can still affect the thorax and diaphragm, inducing changes in respiratory mechanics even when the lungs are healthy, due to increased respiratory effort and compromised gas transport system (9-11). Overweight may also determine the hypertonia of the abdominal muscles and thus compromise respiratory function depending on the diaphragmatic action (11). However, patients with COPD present functional changes in cardiac autonomic modulation, such as reduction of the baroreflex sensitivity, decreased heart rate variability (HRV), and respiratory sinus arrhythmia (12). In addition, patients with hypoxemia (such as COPD) present changes in peripheral chemoreflexes and show increased sympathetic activity (13). Furthermore, decreases in the quality of life are a common problem in these patients due to conditions such as depression, anxiety, and poor sleep quality (14). These factors can also negatively influence the autonomic modulation of individuals with chronic diseases or not (14).

When pulmonary function impairment is established by COPD and is associated with obesity, the state of complexity and autonomic imbalance increases the incidence of comorbidities, such as arterial hypertension and obstructive sleep apnea syndrome (15). Thus, this study aimed to compare the autonomic modulation and sleep behavior of eutrophic and overweight patients with COPD.

## METHODS

This controlled study included sedentary subjects diagnosed with COPD recruited at the Pulmonary Rehabilitation Program between 2016 and 2017 at the University Hospital “Presidente Dutra” in the city of São Luis-MA, in northeastern Brazil. Subjects with COPD were classified into two groups: eutrophic and overweight. They were divided according to their body mass index (BMI) classification. The eligibility criteria for the eutrophic groups were as follows: (a) subjects with a diagnosis of COPD, (b) stable pharmacological treatment (i.e., use of bronchodilators, did not receive inotropic agents), and (c) BMI<25 kg/m^2^ according to the World Health Organization (WHO) general formula (16). For the overweight group, the criteria were as follows: (a) subjects with a diagnosis of COPD, (b) stable pharmacological treatment (i.e., use of bronchodilators, did not take inotropic agents), and (c) BMI over 30 kg/m^2^ according to the WHO general formula (16). The COPD diagnosis was based on the Brazilian guidelines for COPD (17). All methods in this study were approved by the Institutional Ethics Review Board and followed the Helsinki guidelines. The study was registered in the Brazilian Registry of Clinical Trials, with the number RBR-4mz6w9.

The procedures for the measurement of BP were made according to the guidelines of the Seventh Report of the Joint National Committee on Prevention, Detection, Evaluation, and Treatment of High Blood Pressure (18). In summary, patients remained in the sitting position in a comfortable chair for 20 min. With an automatic and noninvasive BP monitor (BP710, Omron, Tokyo, Japan), three BP measurements were performed on the right arm, with at least a 2-min interval between each one.

One morning was reserved for data collection of spirometry tests (MicroLoop Spirometer, CareFusion, Yorba Linda, CA, USA) by a qualified technician. Subjects underwent the spirometry test in the sitting position, wearing a nose clip. Each patient underwent forced spirometry to obtain the following parameters: forced vital capacity (FVC), forced expiratory volume in 1s (FEV1) as well as the ratio of FEV1 to FVC (FEV1/FVC, expressed as a percentage), and all the predicted values according to the American Thoracic Society (ATS) (19).

In addition to the automatic evaluation performed by the software device, the quality of spirometry tests was assessed according to some criteria, including the number of acceptable maneuvers according to ATS, ranging from 0 to 3, the highest kept by the spirometry software and the reproducibility (FEV1 and FVC were considered reproducible according to ATS criteria when the best two trials differed by not more than 200 mL).

A blinded investigator performed the measurements of maximal respiratory pressure. A pressure transducer (MVD-300, Globalmed, Porto Alegre, Brazil) connected to a system with two unidirectional valves (DHD Inspiratory Muscle Trainer, Chicago, IL, USA) was used. The maximal inspiratory pressure (PI_max_) and maximal expiratory pressure (PE_max_) were determined during deep inspiration and expiration from the residual volume against an occluded airway with a minor air leak (2 mm), following a previously described procedure (20). The highest pressures of six measurements were defined as PI_max_ and PE_max_.

The RR interval waves were continuously recorded for 20 min in individuals in supine position, using an electrocardiogram Wincardio (600 Hz) (Micromed, Brazil) for spectral analysis of the HRV. The spectrum resulting from the fast Fourier transform modeling is derived from all data present in the recorded signal; it includes the entire signal variance, regardless of whether its frequency components appear as specific spectral peaks or as nonpeak broadband powers.

The RR interval variability was evaluated in the time (total variability [ms^2^], standard deviation between normal to normal heart beats (SDNN) [ms], and root mean square of successive differences between normal heart beats (RMSSD) [ms]), frequency (low [LF; ms^2^ and %], high [HF; ms^2^ and %], and LF/HF), and nonlinear (SD1 and SD2) domains. The spectral power for LF (0.03-0.15 Hz) and HF (0.15-0.4 Hz) bands was calculated employing power spectrum density integration within each frequency bandwidth through the fast Fourier transform, using the Kubios analysis software (Kubios HRV Standard, V 3.0, Finland) (21).

The six-minute walk test (6MWT) was conducted according to the guidelines established by the ATS. Considering a possible learning effect, the test was performed at screening and habituation visits (22).

Data are reported as mean±standard deviation. Normality of data was tested using the Kolmogorov-Smirnov test. An analysis of covariance (ANCOVA) was conducted to adjust for cofactors. For the comparison of means, the unpaired t-test was used. Differences were considered significant at the 5% level of significance (*p*<0.05). Statistical analysis was performed using GraphPad Prism version 5 (GraphPad Software, San Diego, CA, USA).

Effect size values higher than 0.8 were considered very strong; values ranging from 0.6 to 0.8 were considered strong; values ranging from 0.4 to 0.6 were considered moderate, and values lower than 0.4 were considered negligible (23).

## RESULTS

In this comparative study, 24 COPD patients participated in the analyses of HRV and sleep quality. The difference between the groups was evidenced by BMI classification, where the results denote the difference in weight and height between the groups. No differences were found between the groups for age, sistolic blood pressure (SBP), diastolic blood pressure (DBP), and 6 min walk test (6MWT). However, there was a strong effect size (ES) in the SBP (*p*<0.05, ES=1.05) and body mass index (BMI) (*p*<0.05, ES=3.45) in the overweight group compared to that in the eutrophic group (Table 1).

As shown in Table 1, biochemical markers of patients in the eutrophic and overweight groups, such as the hemoglobin, hematocrit, urea, and calcium levels of the groups, were similar. However, the glycemic levels were higher in the overweight group than that in the eutrophic group. In the evaluation of the anxiety and depression questionnaires, the two groups presented similar scores indicating a mild level of anxiety and a moderate level of depression.

Regarding lung function, the spirometry variables did not differ between the groups in analyzed variables (Table 2). This shows that the groups were homogeneous.

**Figure 1:**
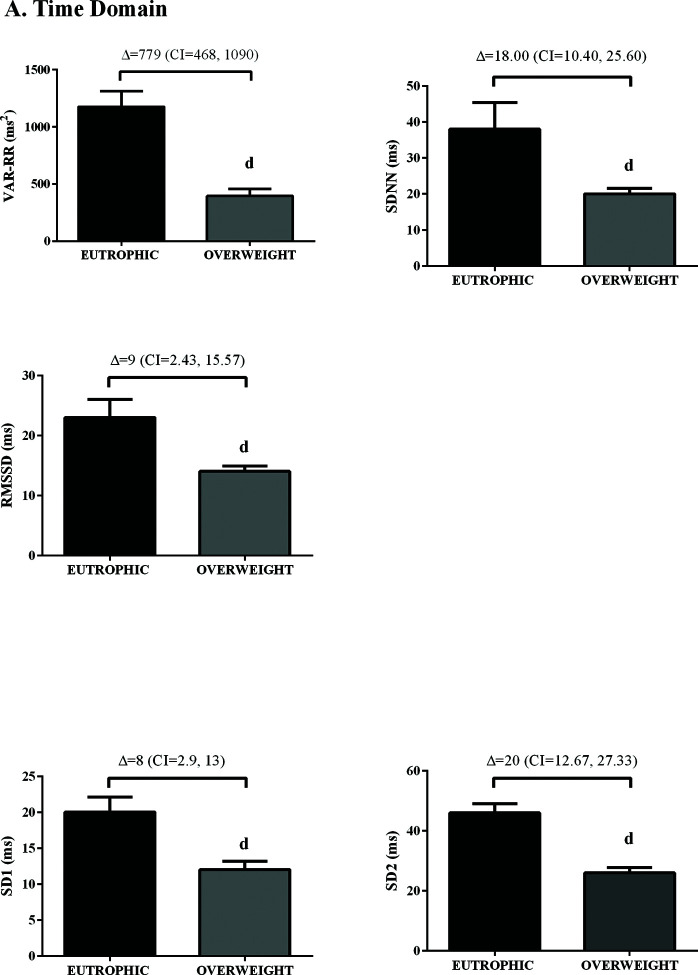
Values are presented as mean±standard deviation or adjusted mean difference (95% confidence interval). Unpaired *t*-test; d*p*<0.05 *vs.* euthrophic.

**Figure 2:**
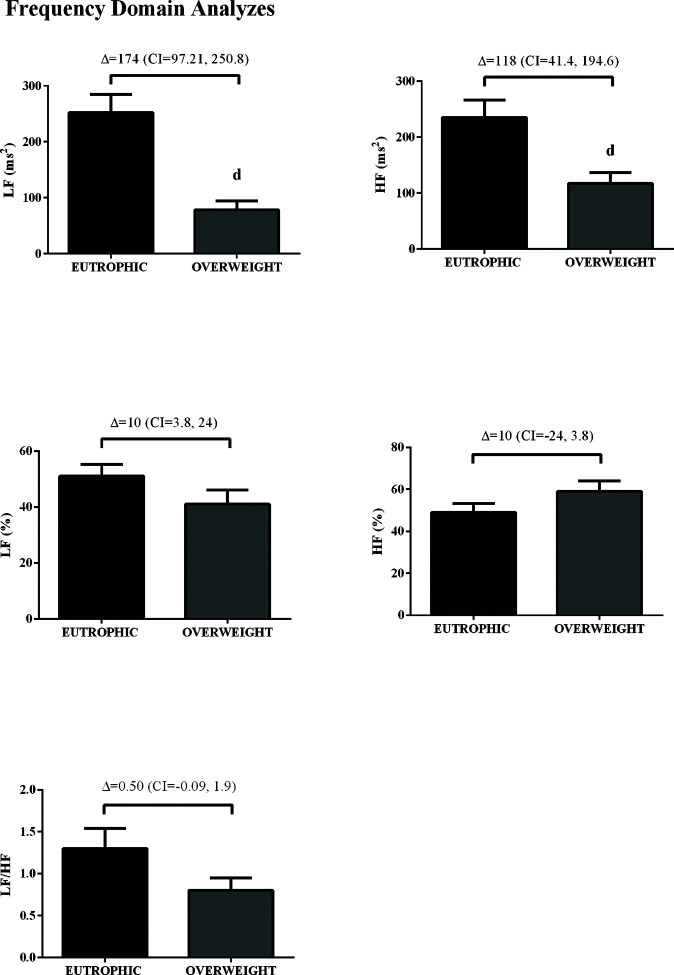
Values are presented as mean±standard deviation or adjusted mean difference (95% confidence interval). d*p*<0.05 *vs.* euthrophic.

In the time domain, the indices of total variability (*p*<0.05, ES=-0.99) and SDNN (*p*<0.05, ES=-1.16) were lower in the overweight group, demonstrating a change in behavior in sympathetic modulation. In addition, reduced RMSSD values were observed in the overweight group (*p*<0.05, ES=0.50) (Figure 1). In addition, in the HRV frequency domain, the LF (ms^2^) and HF (ms^2^) components had lower values in the overweight group (Figure 2). However, no difference was observed in the other parameters in the frequency domain.

In Table 3, we highlight that the three main variables analyzed by the Pittsburgh Sleep Quality Index indicate that the overweight group had overall sleep impairment, as well as latency, efficiency, and sleep quality damage as related to eutrophic group (*p*<0.05).

## DISCUSSION

The present study demonstrates that overweight COPD patients have ANS imbalance associated with increased sympathetic modulation, reduced vagal modulation, and increased systolic BP. It was also observed that sleep quality was associated with sleep disturbances, as well as increased latency and decreased sleep efficiency in the group of overweight COPD patients compared with that in eutrophic COPD subjects. However, in this study, both overweight and eutrophic patients did not show reduced exercise tolerance to the 6MWT, as already demonstrated by our previous publication (24).

In our study, the stratification of the groups was based on the BMI, since it is already well established in the literature that there is a correlation between body composition and prognosis in COPD (25). Thus, COPD patients with increased BMI or cachexia have worse prognosis (25). It has been shown that over time, patients with COPD have weight gain associated with loss of up to 40% of lean mass (26). This distortion in the body composition of this population is due to the fact that metabolism is the most efficient process, since that the energy stored by the adipose tissue is twice as fast as the energy stored by the skeletal muscles. The predominant anabolic metabolism in muscle contraction leads to the rapid depletion of the energy reserve of this site, it becomes evident why this pathological condition favors the accumulation of body fat (27).

This weight gain due to the accumulation of visceral or regional fat is an important risk factor for the development of other pathologies, as well as sleep negative changes (28) because it favors changes in oxidative stress (8,29), inflammatory profile (29), and impairment of the ANS (30). The association of obesity with sleep quality and behavior was also observed in this study from the Pittsburg questionnaire. This relationship draws our attention because this patient may be affected by obstructive sleep apnea syndrome, aggravating the incidence of dyspnea, which is also related to autonomic cardiac dysfunction (28).

In our results, the statistical analysis of the autonomic modulation demonstrated that time domain (by the RMSSD and SDNN) indexes did not differ between the groups as well as the nonlinear analysis, SD1 and SD2, in contrast with previous findings (31). However, by spectral analysis, *i.e.*, in the frequency domain, LF band was increased in the overweight group, indicative of increased cardiac sympathetic modulation, while vagal modulation, HF band, was reduced in relation to the eutrophic group. In this context, autonomic dysfunction was demonstrated in a study by de Carvalho et al. (32), in which a reduction in the geometric indexes of HRV in individuals with COPD was found, indicating a reduction in the overall HRV (32). Bronchoconstriction in COPD patients is mainly driven by parasympathetic stimuli (33) and is commonly treated by β2-adrenergic receptor agonists, such as salbutamol/albuterol (34). However, previous studies have correlated reduced overall variability (a sign of increased sympathetic modulation) with airway inflammation present in COPD patients (31,35). Therefore, autonomic imbalance, characterized by increased cardiac sympathetic and decreased vagal (parasympathetic) modulation present in COPD patients is still a risk factor for cardiovascular events.

Additionally, recent studies have investigated weight change and daily sleepiness, evidencing that increases in body weight negatively increase daytime sleepiness, mainly through different pathways of obstructive sleep apnea (36), providing evidence and understanding of the relationship between overweight and excessive daytime sleepiness. Furthermore, this lower quality of sleep increases the risk for overweight and, consequently, risk factors such as decrease in leptin levels and insulin sensitivity (37,38). Nevertheless, sleep quality can be a determining factor for regulatory aspects of the ANS as well as its metabolism (37). In addition, clinical factors may be considered when observing that sleep quality or duration may be associated with increased cardiovascular risk factors. Recent data show that sleep duration ≤5h per night is associated with a significantly increased risk of hypertension (37). Our data showed that systolic BP was lower in the eutrophic group than overweight group, a situation that may be related to a vagal component that is decreased in the overweight group, reducing the cardioprotective action of the individuals in this group.

**Figure 3:**
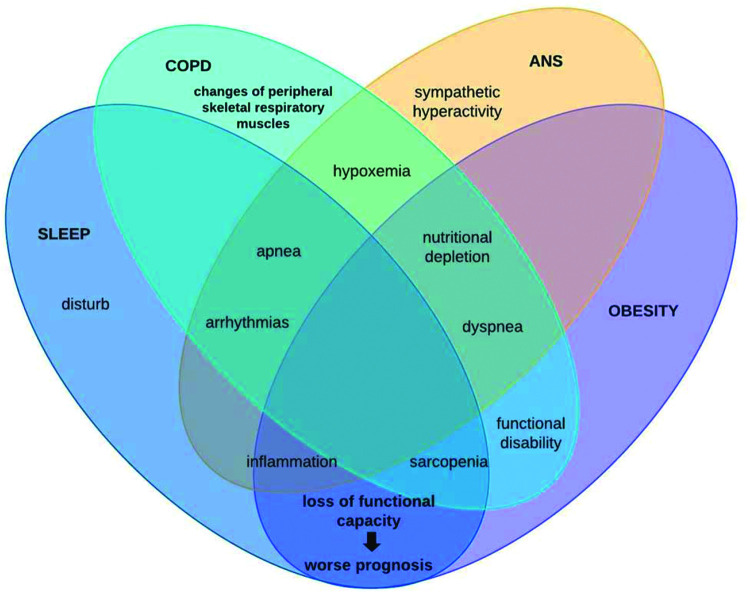
Graphic summary of the relationship between chronic obstructive pulmonary disease (COPD), autonomic nervous system (ANS), obesity, and sleep.

In this context, patients with COPD can be stratified by excessive weight gain or loss. However, it is known that with advancing age, regardless of the increase or reduction, the body composition of elderly individuals is altered through muscle protein synthesis, contributing to sarcopenia genesis (39), remembering that this physical worsening is very prevalent in patients with COPD and, when added to obesity, leads us to reflect on chronic inflammation (28). This condition may be associated with autophagy-related gene expression due to increased expression of toll-like receptors and oxidative stress (40). Conversely, COPD patients with sarcopenia is associated with excessive weight loss, whose main mechanism of muscle atrophy is sustained activation of the atrophy-related genes that reflects persistent cellular stress (40). Therefore, it is noteworthy that the loss of cardiac autonomic complexity, characterized by the variance expected according to the need for body homeostasis, is usually reported in this population, both due to loss of lung function and body weight gain, both with outcome in the systemic inflammatory condition of these patients. Thus, the hypothesis supported by some authors is that in COPD, hyperinflation (40) influences vagal impulses, leading to a reduction in both sympathetic and parasympathetic activity, indicating a worsening sympathovagal balance and, consequently, a worse prognosis associated with adverse clinical events (40). In conclusion, these findings add evidence to the integrative behavior and complications of patients under these conditions. This is because, as shown in Figure 3, autonomic dysfunction impaired by chronic inflammation that obesity establishes interferes with sleep behavior by increasing symptoms of pulmonary dysfunction, leading to a higher prevalence of the COPD, obesity, and OSAS triad, which in turn induces sympathetic hyperactivity, further impairing autonomic modulation, establishing a vicious cycle of dysfunctions.

Thus, our results showed that overweight COPD patients presented lower autonomic modulation and sleep impairment than their eutrophic counterparts, indicating a significant risk factor for cardiovascular diseases and worsening of COPD prognostics.

## AUTHOR CONTRIBUTIONS

Mostarda C and Rodrigues B conceived and designed the analysis, performed the analysis and wrote the manuscript. Barboza CA performed the analysis and wrote the manuscript. Cutrim AL, Silva-Filho AC, Dias CJ and Brito-Monzani JO collected the data, performed the analysis and were responsible for the analysis tools.

## Figures and Tables

**Table 1:** Table of characterization of eutrophic and overweight patients with COPD.

	Eutrophic (n=14)	Overweight (n=10)	Adjusted difference^a^ (95% CI)	ES	*p*
Age (years)	67.93±8.53	68.70±7.68	1.38 (-5.32, 8.08)	0.10	0.8225
Male/female	2/12	2/12	-	-	-
SAP (mmHg)	135.93±14.85	141.7±17.20	13.23 (0.33, 26.14)^b^	1.05^c^	0.3889
DAP (mmHg)	81.43±15.41	77.10±7.08	-1.48 (-13.56, 10.89)	-0.36	0.4184
HR (BPM)	73±14	70±11	-3 (-8.041, 14.04)		0.5788
Weight (kg)	50±5	72±10	22 (-28.41, -15.59)		<0.0001^d^
Height (cm)	151±7	154±3	3 (-7.906, 1.906)		0.2179
BMI (kg/m^2^)	21.60±2.10	29.30±2.35	7.41 (5.59, 9.23)^b^	3.45^c^	<0.0001^d^
Biochemical profile					
Calcium (mg/dL)	9.61±0.30	9.82±0.11	0.19 (-0.16, 0.54)	0.93^c^	0.0471^d^
Glycemia (mg/dL)	94.17±26.27	144.23±8.45	63.77 (36.81, 90.71)^b^	1.83^c^	0.0002^d^
Hematocrit (%)	42.51±4.98	42.30±5.35	-0.77 (-8.10, 6.56)	-0.04	0.9222
Hemoglobin (mg/dL)	14.00±1.65	14.00±1.92	0.14 (-2.37, 2.64)	0.00	1.0000
Urea (mg/dL)	37.75±11.76	37.50±14.08	-4.48 (-27.91, 18.95)	-0.02	0.9627
6MWT (m)	393.61±87.16	355.80±111	32.60 (-46.20, 11.42)	-0.38	0.3597
Subjective analysis					
Sleep quality	5.57±2.40	11.40±3.67	5.82 (3.41, 8.17)^b^	0.71^c^	0.0001^d^
BAI	18±10.21	16±11.01	-0.36 (-1.50, 0.79)	-0.19	0.6514
BDI	21±14	19±12	-0.60 (-2.00, 0.80)	-0.15	0.7183

Values are presented as mean±standard deviation or adjusted mean difference (95% confidence interval). ES, effect size; ^a^ANCOVA adjusted for age, weight, and baseline; unpaired t-test to compare means. ^b^significant difference (*p*<0.05); ^c^clinically relevant (ES>0.80); ^d^ *p*<0.05 *vs.* eutrophic. SAP, systolic arterial pressure; DAP, diastolic arterial pressure; HR, heart rate; BMI, body mass index; 6MWT, six-minute walk test distance; BAI, Beck Anxiety Inventory; BDI, Beck Depression Inventory.

**Table 2:** Respiratory variables between eutrophic and overweight COPD patients.

	Eutrophic (n=14)	Overweight (n=10)	Adjusted difference^a^ (95% CI)	ES	*p*
PI_max_ (cmH_2_O)	-60.71±20.86	-58.70±12.38	5.07 (-9.83, 19.97)	0.12	0.7886
PE_max_ (cmH_2_O)	79.64±28.44	81.50±32.87	-8.12 (-37.71, 21.47)	-0.06	0.8836
FVC (L)	2.21±0.67	1.96±0.61	-0.42 (-0.90, 0.05)	-0.40	0.3602
FEV_1_ (L)	1.01±0.50	1.32±0.43	0.33 (-0.04, 0.70)	0.65	0.3602
FEV_1_/FVC (%)	45.73±10.96	52.59±11.06	5.86 (-1.92, 13.65)	0.62	0.1274
FEF_25-75%_	0.53±0.21	0.69±0.49	0.16 (-0.23, 0.56)	0.42	0.1463

Values are presented as mean±standard deviation or adjusted mean difference (95% confidence interval). ES, effect size; ^a^ANCOVA adjusted for age, weight, and baseline; Unpaired t-test to compare means; PI_max_, maximal inspiratory pressure; PE_max_, maximal expiratory pressure; FVC, forced vital capacity; FEV1, forced expiratory volume in 1s; FVC/FEV1, ratio of FEV1 to FVC.

**Table 3:** The Pittsburgh Sleep Quality Index.

		Eutrophic (n=14)		Overweight (n=10)		
Variable	Category	N	%	N	%	*p*
Subjective sleep quality	Very good	1	7%	0	0%	0.09
	Good	11	79%	4	40%	
	Bad	1	7%	5	50%	
	Too bad	1	7%	1	10%	
Sleep latency	≤15	10	71%	1	10%	0.02^d^
	16-30	0	0%	2	20%	
	31-60	2	14%	3	30%	
	>60	2	14%	4	40%	
Duration of sleep	>7	7	50%	2	20%	0.2
	06/jul	5	36%	3	30%	
	05/jun	1	7%	1	10%	
	<5	1	7%	4	40%	
Sleep efficiency	>85	8	57%	1	10%	0.04^d^
	75-84	5	36%	5	50%	
	65-74	1	7%	1	10%	
	<65	0	0%	3	30%	
Sleep disorders	None	1	7%	0	0%	0.1
	<1	6	43%	1	10%	
	01/fev	7	50%	7	70%	
	3	0	0%	2	20%	
Use of sleeping medication	None	13	93%	8	80%	0.2
	<1	1	7%	0	0%	
	01/fev	0	0%	0	0%	
	3	0	0%	2	20%	
Daytime sleepiness	None	5	36%	1	10%	0.2
	Small	5	36%	3	30%	
	Moderate	4	29%	4	40%	
	Much	0	0%	2	20%	
Sleep quality	Good (0-4)	5	36%	0	0%	0.01^d^
	Bad (5-10)	9	64%	4	40%	
	Disturb (>10)	0	0%	6	60%	

Values are presented in absolute and percentual values. d*p*<0.05 *vs*. eutrophic.
